# The association between built environment features and physical activity in the Australian context: a synthesis of the literature

**DOI:** 10.1186/s12889-016-3154-2

**Published:** 2016-06-08

**Authors:** Belen Zapata-Diomedi, J. Lennert Veerman

**Affiliations:** The University of Queensland, School of Public Health, Herston, QLD 4006 Australia; Centre for Research Excellence in Healthy, Liveable Communities, c/- McCaughey VicHealth Community Wellbeing Unit, Melbourne School of Population and Global Health, Melbourne University, Bouverie Street, Parkville, VIC 3010 Australia; Centre for Research Excellence in Obesity Policy and Food Systems, c/- School of Health and Social Development, Deakin University, Burwood Highway, Burwood, VIC 3125 Australia

**Keywords:** Built environment, Physical activity, Australia, Association, Review, Health, Policy

## Abstract

**Background:**

There is growing evidence indicating that the built environment is a determinant of physical activity. However, despite the well-established health benefits of physical activity this is rarely considered in urban planning. We summarised recent Australian evidence for the association built environment-physical activity among adults. This summary aims to inform policy makers who advocate for the consideration of health in urban planning.

**Methods:**

A combination of built environment and physical activity terms were used to systematically identify relevant peer reviewed and grey literature.

**Results:**

A total of 23 studies were included, providing 139 tests of associations between specific built environment features and physical activity. Of the total, 84 relationships using objective measures of built environment attributes were evaluated, whereas 55 relationships using self-reported measures were evaluated. Our results indicate that walkable neighbourhoods with a wide range of local destinations to go to, as well as a diverse use of land, encourage physical activity among their residents.

**Conclusions:**

This research provides a summary of recent Australian evidence on built environments that are most favourable for physical activity. Features of walkability and availability of destinations within walking distance should be accounted for in the development or redevelopment of urban areas. Our findings emphasise the importance of urban planning for health via its impact on population levels of physical activity.

**Electronic supplementary material:**

The online version of this article (doi:10.1186/s12889-016-3154-2) contains supplementary material, which is available to authorized users.

## Background

Physical inactivity is a significant public health concern given the detrimental impact on population health. For example, low levels of physical activity have been associated with higher all-cause mortality [1] and mortality and morbidity of chronic diseases [2, 3]. In Australia, less than 50 % of the adult population meets the recommended physical activity guidelines [4]. Physical inactivity across the Australian adult population is responsible for 6, 8, 10, 11 and 10 % respectively of the burden of coronary heart disease, type 2 diabetes, breast cancer, colon cancer and all-cause mortality [3]. It has also been suggested that inactive lifestyles are related to poorer mental health outcomes [5, 6], falls in older people [7], and higher risk of overweight [8]. Given the detrimental impact of physical inactivity on population health, much emphasis is placed on ways to improve physical activity behaviours.

Researchers acknowledge that to positively change physical activity behaviours across populations, holistic approaches that consider individual as well as environmental interventions are needed. For example, Sallis and colleagues [9] proposed an ecological framework for ‘active living’ which identifies a number of environmental features and their influence on physical activity behaviours. The built environment is the overarching term used in the literature to describe those objective and subjective features in the physical setting in which people spend their time [10]. According to the World Health Organization (WHO), the built environment incorporates the building and transportation design of a city, including factors such as open green spaces, bike ways/sidewalks, shopping centres, business complexes, and residential accommodation [11]. In recent years, the literature assessing the association between the built environment and physical activity outcomes has grown, mainly in the developed world. This includes a number of survey studies assessing correlations between built environment features and physical activity and obesity [12–19] as well as reviews of reviews [20–22].

In this research we reviewed evidence for the association between built environments and physical activity in the Australian context, with the aim of giving an indication of which environmental factors stand out as being related to physical activity. The review was prepared for the Centre of Population Health (CPH) of the Government of New South Wales (Australia) to assist in decision-making regarding the inclusion of physical activity in urban planning. The objective was to summarise the evidence in Australia from 2009 to date for the association between built environment attributes and adult (≥18 years) physical activity.

## Methods

### Search strategy, data sources and inclusion criteria

One author (BZD) systematically searched peer-reviewed and ‘grey’ literature, in English, restricted to human subjects from 2009 onwards limited to Australia. The scope of the review was defined by the CPH in collaboration with the authors to reflect recent context specific evidence for the associations between built environment characteristics and physical activity. Search strategies were defined in collaboration with members of the CPH and applied to both academic datasets and the grey literature (Additional file 1: Appendix A). The following academic databases were systematically searched: Web of Science, Scopus, EBSCOHost (which includes Business Source Complete, CINAHL, MEDLINE, SportDiscus and Econlit), GeoRef and Leisure Tourism. Google was used to search for Government reports and experts in the field were consulted to ensure that all relevant literature was included. Reporting was based on PRISMA guidelines [23] (Additional file 1: Appendix B). Studies included in selected reviews were assessed against the inclusion criteria (see Table 1).

**Table 1:** Inclusion criteria

Criteria
1. Published in English from 1 January 2009 to 15 March 2015
2. Study conducted in the Australian context
3. Primary study or review
4. Presented evidence on the direct association between built environment features and physical activity
5. Adult population (≥18 years)

Studies that compared physical activity behaviours before and after relocation into a different neighbourhood without direct association to a particular built environment attribute were excluded. Studies assessing mediating variables in the association between built environment attributes and physical activity were excluded when a direct association was not provided. Only studies targeting the adult general population were included (see Table 1).

### Built environment attributes

We grouped built environment features into one of seven categories, including five of the “6 Ds” proposed by Ewing and Cervero [24], plus safety, and aggregated neighbourhood measures (see Table 2). We subdivided broad categories (e.g. design and destinations) given the heterogeneity of measures included in them [25]. Features included in each category are presented in Table 2 with the expected direction of the association based on past literature [26–28].

**Table 2:** Categorisation of built environment attributes

Category^a^	Built environment attributes	Expected direction of association
Density	Population density/jobs density	Positive
Diversity	Land use mix/non-residential zone	Positive
Design	Street Network: street connectivity/few cul de sacs/space syntax measures (e.g. local and control integration)/traffic slowing devices/pedestrian crossing/active transport route options/3/4 or more ways intersections	Positive
	Road traffic volume/busy roads	Negative
	Transport infrastructure: sidewalks/bikeways/street lights/aesthetics and attractiveness	Positive
	Green and recreational space: area/presence/number/distance(shorter)/quality/attractiveness/maintenance/aesthetics	Positive
Destination	Transport related: shorter distance (or access within walking distance) to: neighbourhood destinations, retail, school/better job accessibility by public transport	Positive
	Job accessibility by car	Negative
	Recreation related: shorter distance (or access within walking distance) to recreational destinations	Positive
Distance to transit	Shorter distance (or access within walking distance) to bus stops/train stations	Positive
Safety	Neighbourhood lighting	Positive
	Crime/Traffic	Negative
Aggregated neighbourhood characteristics	Walkability index/environmental score	Positive

^a^Note: Ewing and Cervero have a 6th D relating to the Demand for Parking. It has been excluded in this list as no relevant research was found

### Coding of evidence

Most of the studies tested multiple associations as a result of different domains of physical activity assessed, outcomes evaluated, neighbourhood definitions, and spatial area evaluated. Similar approaches were taken in past studies [14, 26]. Results were coded in terms of whether the associations between built environment attributes and physical activity behaviours were in the expected direction (+), in the opposite direction (-), or not statistically significant (0) according to the level of significance stated in the study. We present results for studies in which built environment attributes were both objectively measured and subjectively measured. We also report physical activity based on perceptions of the purpose (e.g. transport and recreational) and total physical activity. Total physical activity was explicitly derived in most of the studies [29–35] as any physical activity in the transport and recreational domains. However, in two studies total physical activity included households and gardening chores [34, 35] (detail can be found in Additional file 1: Appendix D).

We considered objective measures of built environment attributes as showing *sufficient* evidence if they were assessed in at least three independent studies [15]. Of the built environment categories with sufficient evidence, it was deemed *convincing* if at least 50 % of all associations were in the expected direction [15, 26]. Self-reported built environment attributes showing convincing evidence (≥50 % associations in the expected direction from at least 3 independent studies) are presented to assess whether they support objective findings.

### Quality assessment

We assessed the quality of studies (see Additional file 1: Appendix F, Tables 1 and 2) using tools from a similar review [12]. The quality assessment focused on the representativeness of the sample, measurement of outcome variables, and control for confounding variables. Longitudinal (*n* = 2) and quasi-experimental designs (*n* = 2) were assessed separately from cross-sectional designs (*n* = 19). Studies were classified as being of poor, fair, or good quality according to the number of criteria met. We assessed the strength of the associations with and without quality assessment, following recommendations in the literature to not rely on ‘vote counting’ techniques [36].

## Results

**Fig. 1:**
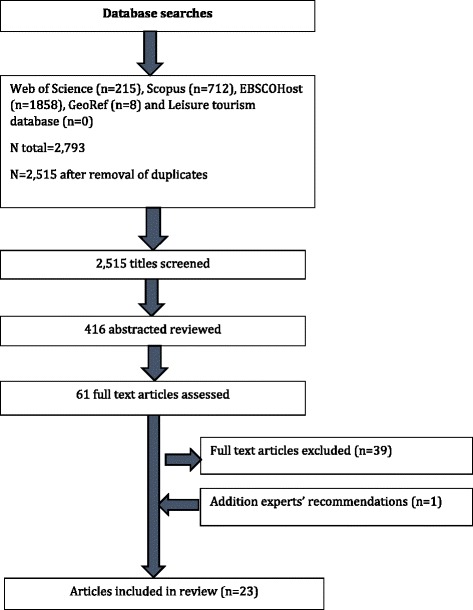
Summary of search results

A total of 22 studies from the database search and one additional study recommended by experts in the field provided 139 associations of built environment attributes and physical activity (Fig. 1). Of the total, 84 associations were evaluated against objective measures of built environment attributes and 55 associations were evaluated against subjective measures (Table 3). A list of excluded papers and reasons for exclusion is provided in Additional file 1: Appendix C.

**Table 3:** Summary of associations between built environment attributes and physical activity

Built environment attributes	Objective built environment		Self-reported built environment	
	All studies	Good and fair quality^b^	All studies	Good and fair quality
Density	3/9 (33 %) [4]^a^	1/5 (20 %) [2]		
Diversity	4/6 (67 %) [3]	2/4 (50 %) [1]		
Design	8/29 (28 %) [6]	6/24 (25 %) [4]	16/32 (50 %) [3]	11/27 (40 %) [3]
Destinations	7/10 (70 %) [4]	3/6 (50 %) [2]	10/14 (71 %) [3]	10/14 (71 %) [3]
Distance to transit	4/5 (80 %) [3]	3/4 (75 %) [2]	1/2 (50 %) [1]	1/2 (50 %) [1]
Safety	2/6 (33 %) [2]	0/0 (N/A)	3/9 (33 %) [3]	2/9 (22 %) [3]
Aggregated neighbourhood measures	14/19 (74 %) [3]	8/15 53 % [2]	1/1 (100 %) [1]	1/1 (100 %) [1]

Note: Results represent the proportion (%) of tested associations with results in the expected direction

^a^Number of independent studies

^b^Only two cross-sectional design studies rated as good quality, ten qualified as fair quality and seven as poor. All studies with longitudinal and quasi-experiment designs rated as good quality (Additional file 1: Appendix F Tables 1 and 2)

### Study characteristics

The largest proportion of studies was conducted in Western Australia (*n* = 8), followed by South Australia (*n* = 7), New South Wales (*n* = 3), Victoria (*n* = 3) and Queensland (*n* = 2) (Table 1, Additional file 1: Appendix D). Most of the studies were cross-sectional in design (*n* = 19), with two longitudinal studies [37, 38] and two quasi-experiments [39, 40]. The median response rate across studies reporting it was 31 %, ranging from 11.5 % [38, 41–44] to 68.5 % [29]. The majority of included studies randomly selected participants, with the exception of five studies from the RESIDE project which selected participants according to their intention to relocate to new developments [31, 33, 40, 45, 46]. The median sample size for studies reporting it was 2194 with a range from 320 individuals [47, 48] to 203,883 individuals [34, 35]. All included studies were from urban areas with one exception for rural zones [49]. For the studies that reported participants’ ages, averages across studies ranged from 35 [49] to 61 [34, 35] years with a mean of 45 years. The older participants were selected only if they were 45 years old or above. The majority of the studies included both genders, with one exception that only included women [49]. For the studies that reported gender distribution, women represented on average 55 % of the samples across the included studies, with the highest proportion at 62 % [32]. Only one study sampled individuals from a specific income group (low socio-economic status) [49].

### Physical activity measures

All included studies used self-reported measures of physical activity for a usual week (i.e. previous seven days/week), or the past month. Walking was the most commonly assessed physical activity outcome (*n* = 16), followed by cycling (*n* = 3) [43, 45, 50], moderate to vigorous physical activity (*n* = 3) [34, 35, 44], leisure time physical activity (*n* = 1) [49], and use of active travel modes (*n* = 1) [51]. One study assessed both walking and moderate to vigorous physical activity [35]. In less than half of the included studies (*n* = 9) [31, 33, 39, 40, 44–48] physical activity was measured using questionnaires that specified the location (e.g. neighbourhood) in which activities took place.

### Built environment measures

All studies, except one [51], assessed built environment attributes in the neighbourhood area, commonly defined as the 1.6 km street network service area, or 1 km radius from a participant’s residence, or walking area within 10 to 15 min. Ten studies used objective measures of built environment attributes, eight studies used both objective and subjective measures, and five studies used only subjective measures.

### Summary of findings

**Fig. 2:**
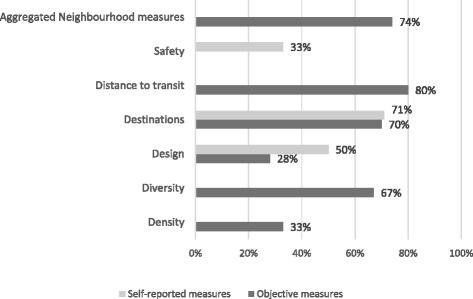
Proportion of tested associations for built environment features with sufficient evidence in the expected direction

The greatest number of associations that were evaluated against objective measures of the built environment addressed total physical activity (*n* = 32), physical activity related to transport (*n* = 28), and leisure specific physical activity (*n* = 24). In the following section and in Fig. 2 and Table 3, we present a summary of the evidence with complete results for each physical activity domain and subcategories of the built environment attributes available in Additional file 1: Appendix E.

#### Density

After adjustment for other explanatory variables, the evidence for the effect of density on physical activity outcomes is not convincing. For the Australian context, only 33 % of cases indicated a positive association of density with physical activity.

#### Diversity

The findings indicate convincing evidence of a positive relationship between built environment diversity measures and physical activity, with four out of six studied associations in the expected direction (67 %). This indicates that greater diversity in the built environment is associated with greater physical activity. All four positive associations for measures of land use mix were related to physical activity in the transport domain.

#### Design

The evidence for the relationship between design features and physical activity outcomes is not convincing, with only 28 % of associations in the expected direction. When assessing the subcategories of design (e.g. street network, transport infrastructure, and green and recreational spaces), the evidence remains unconvincing, or not sufficient to draw conclusions.

#### Destinations

The evidence of a relationship between availability of destinations and physical activity outcomes is convincing with 70 % of the associations showing an effect in the expected direction. The majority (6/8) of the evidence for destination measures relates to transport destinations such as retail zones, services, post offices, food outlets, transit stops, job locations, and open public spaces such as parks. All but one positive association related to transport or total physical activity.

#### Distance to transit

The current evidence provides convincing evidence for the association between shorter distance to transit and physical activity, with 80 % of associations in a positive direction. It should be noted that many of the studies include transit as a measure under ’destinations’. Most of the associations for physical activity measures relate to transport physical activity, with one exception relating to total physical activity.

#### Safety

Two studies indicated that safety is associated with total physical activity. However, in one of the studies the direction of the associations were not in the expected direction, indicating that less safe places were associated with positive physical activity outcomes. The evidence remains inconclusive since we found only two studies and there is no consensus in the potential effect of safer places.

## Aggregated neighbourhood measures

Aggregated neighbourhood measures, such as walkability, are composite indices that include a number of built environment features such as density, connectivity, and land use mix [52]. Convincing evidence was found in the Australian context for aggregated neighbourhood measures with 74 % of the associations indicating a positive impact on physical activity. Walkability measures indicated a stronger association for transport related physical activity (7/7) and total physical activity (5/6) in comparison to physical activity for recreational purposes (2/6).

### Evidence for self-reported built environment attributes

For perceived measures of the built environment, most studies evaluated leisure physical activity outcomes (*n* = 41) and physical activity for transport purposes (*n* = 14) (Additional file 1: Appendix E).

Associations between physical activity and perceived built environment attributes were not always in line with similar relationships that used objective measures. As shown in Fig. 2 and Table 3, there is sufficient evidence to draw conclusions for destinations, safety, and design. For destinations, the evidence was similar to objective measures. On the other hand, the evidence from self-reported measures indicated that built environment attributes related to design are positively associated with physical activity outcomes.

### Sensitivity of results to study quality

If we consider only studies judged to be of good quality, there is not enough evidence to draw conclusions for any of the associations between objectively measured built environment attributes and physical activity outcomes (Table 3). The only exception is measures of design, for which the evidence is sufficient in quantity, however this evidence does not convincingly show an association with physical activity outcomes (25 %). When the components of design are individually analysed, there is convincing evidence for a positive association between the street network subcategory (street connectivity) and physical activity outcomes (50 %). For the case of evidence using self-reported built environment measures, conclusions remained unchanged for all except for measures of design (from 50 to 40 %) after taking the quality of the studies into account.

## Discussion

In this review we summarise the recent Australian literature measuring the association between physical activity and built environment attributes. A total of 23 quantitative studies that focused on adults’ physical activity were reviewed for both objective and self-reported measures of the built environment. As a whole, evidence indicates a positive relationship between built environment attributes and physical activity for adults. Objective measures of built environment attributes that were positively associated with physical activity included destinations within walking or cycling distance from the residence, shorter distance to transportation, such as bus stops, train stations, and ferry terminals, walkability, and higher diversity of land uses. Findings were similar for both objective and self-reported measures of availability of destinations. Although self-reported measures of design indicated convincing evidence of an association with physical activity, this was not the case for objective measures. Both objective and perceived measures of the built environment are considered important as they provide insight into different relationships with physical activity outcomes [36]. For example, a range of social, economic and demographic factors are likely to influence individuals’ perceptions of the built environment, which not necessarily correspond to objective measures [53, 54]. We did not differentiate results in regards to self-reported and objective measures of physical activity as all included studies relied on self-reported measures. However, in our sensitivity analysis we did consider the quality of reporting (i.e. the use of a validated questionnaire). We could not identify a consistent pattern for results when comparing studies using validated questionnaires against those that did not. Nevertheless, four out of six studies not using validated questionaries were classified as being of poor quality according to the criteria used in this study.

For objective measures of design and density, there was not convincing evidence to indicate that these variables are associated with physical activity from recent studies in the Australian context. However, measures of density, connectivity (design feature), and open public spaces (design and destinations feature), are commonly included in aggregated neighbourhood measures, which shows convincing evidence of having a positive relationship with physical activity. Additionally, having more places to visit implies various components of design such as parks and green open spaces. While density itself is unlikely to stimulate physical activity, higher density allows for mass transit and commercial and non-commercial destinations and therefore tends to increase the number of potential destinations within walking or cycling distance [39, 55]. In accounting for these mediating variables, there is a risk of over-adjustment and ‘explaining away’ real associations. Hence, it may be that a mix of built environment attributes is needed to have a positive impact on physical activity. The overall evidence summarised in this review suggests that having access to a wide variety of destinations within walking distance supports higher levels of physical activity.

We reported physical activity outcomes in two domains (recreational and transport) as well as total physical activity following recommendations from the literature regarding the different uses of built environment features for physical activity [36, 56]. However, results should be interpreted with caution. For example, we cannot conclude that diversity is more important for transport physical activity than for recreational physical activity as we do not have the same number of associations across all domains of physical activity.

After excluding studies that did not meet quality criteria, there was insufficient evidence to draw conclusions for any of the objective measures of the built environment except street connectivity (design measures within street network category). The results for self-reported measures remained unchanged, expect for the case of measures of design. Our results highlight the importance of taking the quality of the studies into account in the process of summarising the literature addressing association between the built environment and physical activity. As highlighted in the past, quality assessment of primary studies is rarely carried out in despite of being one of the main criteria of a systematic review [36]. The traditional hierarchical classification of evidence recommended by the National Health and Medical Research Council of Australia (NHMRC) [57] is ill-suited for the quality evaluation of studies on the relationship between the built environment and physical activity. This is because it does not distinguish between different observational designs. It focuses on experiments, which are seldom feasible in this field. We decided to use a tool based on quality criteria for the evaluation of observational studies proposed by Petticrew and Roberts as adapted by Grasser and colleagues [12]. However, we added a criterion to assess whether cross-sectional studies included a measure to control for self-selection, given its attenuating effect for the association built environment and physical activity [19].

Our findings are specific to the Australian context. Nevertheless, they are in line with internationally conducted literature surveys. Recent reviews found that availability of destinations (overlapping with land use mix) and walkability are facilitators of physical activity. McCormack and Shiell [19] conducted a systematic review of the international literature on the association of objectively measured built environment features and physical activity, including only studies that controlled for self-selection (cross sectional controlling for self-selection and quasi-experimental designs). They found consistent associations between physical activity and land use mix, composite walkability indices, and neighbourhood type (i.e. neo-traditional versus conventional). A study focusing only on European countries found convincing evidence for an association between physical activity and walkability, access to shops, services, and work, and environmental quality [15]. Grasser and colleagues [12] found consistent associations between physical activity and density (i.e. population, housing, and intersections) and walkability indices.

Strengths of this study include the systematic review of evidence that is recent and directly applicable to the Australian context, and the ascertainment of study quality. It is worth noting that the inclusion of quality criteria for studies assessing the association between the built environment and physical activity is uncommon in the literature [36]. Furthermore, the search strategy was defined in collaboration with a group of experts in the field and policy makers, as this review is part of a broader review for a government body. Limitations of this study should be mentioned. While a comprehensive search strategy was followed, only one reviewer was in charge of systematically reviewing the literature. However, given that the process was overseen by a group of experts, the potential of missing relevant studies was small. Besides, it can be argued that in the aim of showing recent Australian evidence and limiting the review to 2009 onwards, important literature may have been missed. Furthermore validated physical activity questionnaires were not used in six out of 23 studies which may had resulted in biased results for the assessed associations [58]. We attempted to pool results in a meta-analysis, however, given the diversity in the ways in which different studies report their findings this was not possible. Besides, the greatest majority of the evidence relies on cross-sectional designs, which does not allow for causal inference.

### Recommendations for future research

We observed a number of limitations in the literature that should be addressed in future research assessing the relationship between built environment attributes and physical activity.

1. Use of standard methods for reporting the association between the built environment and physical activity allowing for the statistical combination of results. This may include moving from categorical exposures to continuous measures. As recently suggested by Lamb and White [59], using continuous exposure measures would also avoid the loss of exposure information which occurs in the categorisation process. Pooling results from studies has numerous advantages, including a higher number of observations for a given association and hence greater statistical power and improved estimates of effect size [60].
2. Provide sufficient information on the exposure variable to enable a direct interpretation of results. For example, presenting results in terms of associations of physical activity outcomes with z-scores is meaningless without descriptive information about the distribution of the exposure variable (i.e. mean and standard deviation). Furthermore, the categorisation of exposure variables in quantiles, or ordinal data without an indication of the mean value of each category makes it impossible to know what level of change in the exposure variable is needed to achieve a certain outcome. In plain summary, all we know from the literature is that more is better than less (or vice versa, depending on the exposure), however, we are unsure about how much change is needed. Hence, researchers investigating the potential effect of physical activity of changes in the built environment should be specific in the level of change in the exposure variable (e.g. increase in 8 dwelling per hectare). This is of particular relevance for policy makers who need robust information on what environmental factors are associated with physical activity behaviours and how much of each is needed to achieve meaningful health benefits.
3. Researchers should take into account mechanisms to diminish the potential bias introduced by self-selection such as longitudinal and quasi-experimental designs, or inclusion of question to assess potential self-selection in cross sectional studies. The majority of studies are cross sectional in design, which does not allow for a direct causal interpretation. The association may be due to the built environment influencing physical activity; this is the hypothesis underlying this research. Alternatively, it may be due to physically active people choosing to live in neighbourhoods that facilitate that behaviour. By adjusting for self-selection, some studies try to avoid this ‘reverse causal’ interpretation. McCormack and Shiell [19] systematically reviewed the international literature and found that adjusting for self-selection tended to diminish the strength of the associations, but only to a small extent. Finally, the associations could be due to other (observed or unobserved) factors causing both (i.e., confounders). Most studies use statistical adjustment to minimise the impact of measured factors. However, it is unclear what unobserved factors could explain the associations.

## Conclusions

This is the first review for the built environment correlates of physical activity among adults specific to the Australian context. We found convincing evidence that people who live in neighbourhoods with a large availability of destinations within walking/cycling distance are more likely to engage in physical activity. Objectively measured distance to transit, destinations and land use measures supported this conclusion. Likewise, self-reported measures of destinations and design indicated a positive relationship with physical activity. On the other hand, for objectively measured density and design the evidence of association with physical activity was insufficient or inconclusive. However, this should be interpreted cautiously as without density there would not be people to go to the destinations, and design features such as connectivity enable people to reach destinations. In this review we found that commonly cited correlates of physical activity in international literature also apply to Australia (destinations, diversity and measures of walkability).

This review has also demonstrated that results for objectively measured built environment features differ with those for self-reported measures. Investigating objective and self-reported measures of built environment attributes has been recommended in the literature, as these are likely to relate differently to physical activity outcomes. For example, even though a neighbourhood could be unsafe in terms of objective measures of crime, people may not perceive this and rate crime as a non-issue for being physically active (or vice versa).

Results presented in this review are of use to policy makers in the health sector who advocate for the inclusion of physical activity in urban and transport planning.

## Additional file

Additional file 1: Appendix A: Table 1 Databases search strategy. Table 2 Searches Appendix B: Table 1 Checklist of items to include when reporting a systematic review or meta-analysis. Appendix C: Table 1 Excluded studies. Appendix D: Table 1 Summary of studies’ characteristics for built environment attributes-physical activity association for Australian adults. Appendix E: Table 1 Summary of findings. Appendix F: Table 1 Quality assessment cross sectional studies. Table 2 Quality assessment longitudinal and quasi experiments studies. (DOCX 308 kb)
