# Supplementary material for: The association between built environment features and physical activity in the Australian context: a synthesis of the literature
Source: BMC Public Health. 2016 Jun 8;16:484. doi: 10.1186/s12889-016-3154-2 (PMC4898384; doi:10.1186/s12889-016-3154-2)
Supplement: Additional file 1: — Appendix A: Table 1 Databases search strategy. Table 2 Searches Appendix B: Table 1 Checklist of items to include when reporting a systematic review or meta-analysis. Appendix C: Table 1 Excluded studies. Appendix D: Table 1 Summary of studies’ characteristics for built environment attributes-physical activity association for Australian adults. Appendix E: Table 1 Summary of findings. Appendix F: Table 1 Quality assessment cross sectional studies. Table 2 Quality assessment longitudinal and quasi experiments studies. (DOCX 308 kb) [file 12889_2016_3154_MOESM1_ESM.docx]

**Appendix A**

**Table 1 Databases search strategy**

| **Databases** | **Search terms and their variants** |
| --- | --- |
| Web of Science, Scopus, EBSCOHost (including: Business Source Complete, CINAHL, MEDLINE, SportDiscus, Econlit), GeoRef, Leisure tourism database | **Built environment terms**  spatial OR neighbourhood OR neighborhood OR “built environment” OR “physical environment” OR “street scape” OR “urban form” OR “urban planning” OR walkability OR “pedestrian friendly” OR walkable OR cyclable OR cyclability OR density OR “land use” OR “urban design” OR” open space” OR “green space” OR parks OR “street layout” OR ”public transport” OR” street connectivity” OR pathway OR ”cycle paths” |
|  | **PA terms**  “physical activity" OR exercise OR inactivity OR walking OR bicycling OR strolling OR "leisure time" OR sports OR recreation OR "active transpor*" OR pedestrian OR "active travel" OR "active living" OR "active recreation" OR liveable OR walk OR cycle OR cycling OR bicycle OR "healthy lifestyle" OR obesity OR overweight OR sedentary |

**Table 2 Searches**

|  | **Urban form and economic terms** |
| --- | --- |
|  | **Search strategy** |
| Web of Science | TI=(spatial OR neighbourhood OR neighborhood OR “built environment” OR “physical environment” OR “street scape” OR “urban form” OR “urban planning” OR walkability OR “pedestrian friendly” OR walkable OR cyclable OR cyclability OR density OR “land use” OR “urban design” OR” open space” OR “green space” OR parks OR “street layout” OR ”public transport” OR” street connectivity” OR pathway OR ”cycle paths”) AND TI=(“physical activity" OR exercise OR inactivity OR walking OR bicycling OR strolling OR "leisure time" OR sports OR recreation OR "active transpor*" OR pedestrian OR "active travel" OR "active living" OR "active recreation" OR liveable OR walk OR cycle OR cycling OR bicycle OR "healthy lifestyle" OR obesity OR overweight OR sedentary) AND CU=Australia |
| Scopus | TITLE ( spatial OR neighbourhood OR neighborhood OR "built environment" OR "physical environment" OR "street scape" OR "urban form" OR "urban planning" OR walkability OR "pedestrian friendly" OR walkable OR cyclable OR cyclability OR density OR "land use" OR "urban design" OR " open space" OR "green space" OR parks OR "street layout" OR "public transport" OR " street connectivity" OR pathway OR "cycle paths" ) AND TITLE-ABS-KEY ( "physical activity" OR exercise OR inactivity OR walking OR bicycling OR strolling OR "leisure time" OR sports OR recreation OR "active transpor*" OR pedestrian OR "active travel" OR "active living" OR "active recreation" OR liveable OR walk OR cycle OR cycling OR bicycle OR "healthy lifestyle" OR obesity OR overweight OR sedentary ) AND AFFILCOUNTRY ( australia ) AND REFPUBYEAR > 2008 |
| EBSCOHost (including: Business Source Complete, CINAHL, MEDLINE, SportDiscus, Econlit), | TI ( spatial OR neighbourhood OR neighborhood OR “built environment” OR “physical environment” OR “street scape” OR “urban form” OR “urban planning” OR walkability OR “pedestrian friendly” OR walkable OR cyclable OR cyclability OR density OR “land use” OR “urban design” OR” open space” OR “green space” OR parks OR “street layout” OR ”public transport” OR” street connectivity” OR pathway OR ”cycle paths” ) AND TI ( “physical activity" OR exercise OR inactivity OR walking OR bicycling OR strolling OR "leisure time" OR sports OR recreation OR "active transpor*" OR pedestrian OR "active travel" OR "active living" OR "active recreation" OR liveable OR walk OR cycle OR cycling OR bicycle OR "healthy lifestyle" OR obesity OR overweight OR sedentary ) Limiters - Published Date: 20090101-20151231; English Language; Language: English; English Language; Geographic Subset: Australia & New Zealand; Language: English; Language: English; Country: Australia |
| GeoRef | ((spatial OR neighbourhood OR neighborhood OR "built environment" OR "physical environment" OR "street scape" OR "urban form" OR "urban planning" OR walkability OR "pedestrian friendly" OR walkable OR cyclable OR cyclability OR density OR "land use" OR "urban design" OR" open space" OR "green space" OR parks OR "street layout" OR "public transport" OR" street connectivity" OR pathway OR "cycle paths") wn AB and ("physical activity" OR exercise OR inactivity OR walking OR bicycling OR strolling OR "leisure time" OR sports OR recreation OR "active transpor*" OR pedestrian OR "active travel" OR "active living" OR "active recreation" OR liveable OR walk OR cycle OR cycling OR bicycle OR "healthy lifestyle" OR obesity OR overweight OR sedentary) WN TI) AND ((Australia) WN CO) |
| Leisure tourism database | title:(spatial OR neighbourhood OR neighborhood OR "built environment" OR "physical environment" OR "street scape" OR "urban form" OR "urban planning" OR walkability OR "pedestrian friendly" OR walkable OR cyclable OR cyclability OR density OR "land use" OR "urban design" OR" open space" OR "green space" OR parks OR "street layout" OR "public transport" OR" street connectivity" OR pathway OR "cycle paths") AND title:("physical activity" OR exercise OR inactivity OR walking OR bicycling OR strolling OR "leisure time" OR sports OR recreation OR "active transpor*" OR pedestrian OR "active travel" OR "active living" OR "active recreation" OR liveable OR walk OR cycle OR cycling OR bicycle OR "healthy lifestyle" OR obesity OR overweight OR sedentary) AND yr:[2009 TO 2015] |

**Comments:** the country filters are not accurate; they still include other countries studies which were excluded.

**Grey literature search**

Grey literature, was identified from selected studies references lists and also tracking citing documents using SCOPUS.

Google was searched, using the advanced search function. A combination of terms from table 1 was used and the document type filter was used to only capture pdf files, also the search was limited to and file extensions gov, edu, au and org. The first 100 titles were scanned for relevance using the study inclusion criteria

**Appendix B**

**Table 1 Checklist of items to include when reporting a systematic review or meta-analysis (1)**

| Section/topic | # | | Checklist item | Reported on page # |
| --- | --- | --- | --- | --- |
| **TITLE** | | | | |
| Title | | 1 | Identify the report as a systematic review, meta-analysis, or both. | NA |
| **ABSTRACT** | | | | |
| Structured summary | | 2 | Provide a structured summary including, as applicable: background; objectives; data sources; study eligibility criteria, participants, and interventions; study appraisal and synthesis methods; results; limitations; conclusions and implications of key findings; systematic review registration number. | 1 & 2 |
| **INTRODUCTION** | | | | |
| Rationale | | 3 | Describe the rationale for the review in the context of what is already known. | 2 & 3 |
| Objectives | | 4 | Provide an explicit statement of questions being addressed with reference to participants, interventions, comparisons, outcomes, and study design (PICOS). | 2 & 3 |
| **METHODS** | | | | |
| Protocol and registration | | 5 | Indicate if a review protocol exists, if and where it can be accessed (e.g., Web address), and, if available, provide registration information including registration number. | NA |
| Eligibility criteria | | 6 | Specify study characteristics (e.g., PICOS, length of follow-up) and report characteristics (e.g., years considered, language, publication status) used as criteria for eligibility, giving rationale. | 3, 4 & 5 |
| Information sources | | 7 | Describe all information sources (e.g., databases with dates of coverage, contact with study authors to identify additional studies) in the search and date last searched. | 3, 4 & 5 |
| Search | | 8 | Present full electronic search strategy for at least one database, including any limits used, such that it could be repeated. | Appendix A |
| Study selection | | 9 | State the process for selecting studies (i.e., screening, eligibility, included in systematic review, and, if applicable, included in the meta-analysis). | 3 |
| Data collection process | | 10 | Describe method of data extraction from reports (e.g., piloted forms, independently, in duplicate) and any processes for obtaining and confirming data from investigators. | 3, 4 & 5 |
| Data items | | 11 | List and define all variables for which data were sought (e.g., PICOS, funding sources) and any assumptions and simplifications made. | 4 & 5 |
| Risk of bias in individual studies | | 12 | Describe methods used for assessing risk of bias of individual studies (including specification of whether this was done at the study or outcome level), and how this information is to be used in any data synthesis. | 5 (Quality Assessment) |
| Summary measures | | 13 | State the principal summary measures (e.g., risk ratio, difference in means). | NA |
| Synthesis of results | | 14 | Describe the methods of handling data and combining results of studies, if done, including measures of consistency (e.g., I^2^) for each meta-analysis. | NA |
| Risk of bias across studies | | 15 | Specify any assessment of risk of bias that may affect the cumulative evidence (e.g., publication bias, selective reporting within studies). | NA |
| Additional analyses | | 16 | Describe methods of additional analyses (e.g., sensitivity or subgroup analyses, meta-regression), if done, indicating which were pre-specified. | NA |
| RESULTS | | | | |
| Study selection | | 17 | Give numbers of studies screened, assessed for eligibility, and included in the review, with reasons for exclusions at each stage, ideally with a flow diagram. | 7 (Figure 1) & Appendix C |
| Study characteristics | | 18 | For each study, present characteristics for which data were extracted (e.g., study size, PICOS, follow-up period) and provide the citations. | Appendix D |
| Risk of bias within studies | | 19 | Present data on risk of bias of each study and, if available, any outcome-level assessment (see Item 12). | Not done |
| Results of individual studies | | 20 | For all outcomes considered (benefits or harms), present, for each study: (a) simple summary data for each intervention group and (b) effect estimates and confidence intervals, ideally with a forest plot. | NA |
| Synthesis of results | | 21 | Present results of each meta-analysis done, including confidence intervals and measures of consistency. | NA |
| Risk of bias across studies | | 22 | Present results of any assessment of risk of bias across studies (see Item 15). | NA |
| Additional analysis | | 23 | Give results of additional analyses, if done (e.g., sensitivity or subgroup analyses, meta-regression [see Item 16]). | 9 |
| DISCUSSION | | | | |
| Summary of evidence | | 24 | Summarize the main findings including the strength of evidence for each main outcome; consider their relevance to key groups (e.g., health care providers, users, and policy makers). | 10, 11 & 12 |
| Limitations | | 25 | Discuss limitations at study and outcome level (e.g., risk of bias), and at review level (e.g., incomplete retrieval of identified research, reporting bias). | 12 |
| Conclusions | | 26 | Provide a general interpretation of the results in the context of other evidence, and implications for future research. | 12 & 13 |
| FUNDING | | | | |
| Funding | | 27 | Describe sources of funding for the systematic review and other support (e.g., supply of data); role of funders for the systematic review. | Acknowledgement |

1. Moher D, Liberati A, Tetzlaff J, Altman DG. Preferred reporting items for systematic reviews and meta-analyses: the PRISMA statement. Annals of internal medicine. 2009;151(4):264-9.

**Appendix C**

**Table 1 Excluded studies**

| **Study** | **Reason for exclusion** |
| --- | --- |
| Astell-Burt, T, Feng, X & Kolt, GS 2014, 'Greener neighbourhoods, slimmer people evidence from 246 920 Australians', International Journal of Obesity, vol. 38, no. 1, pp. 156-9. | Focus on body mass index (BMI) outcomes |
| Astell-Burt, T, Feng, X & Kolt, GS 2014, 'Is neighbourhood green space associated with a lower risk of type 2 diabetes evidence from 267,072 Australians', Diabetes Care, vol. 37, no. 1, pp. 197-201. | Focus on diabetes outcomes |
| Brown, G, Schebella, MF & Weber, D 2014, 'Using participatory GIS to measure physical activity and urban park benefits', Landscape and Urban Planning, vol. 121, pp. 34-44. | Does not give an indication of effect, only cross tabulations |
| Carver, A, Timperio, A & Crawford, D 2008, 'Playing it safe: The influence of neighbourhood safety on children's physical activity-A review', Health and Place, vol. 14, no. 2, pp. 217-27. | Focus on children and pre 2009 |
| Christian, H, Giles-Corti, B, Knuiman, M, Timperio, A & Foster, S 2011, 'The influence of the built environment, social environment and health behaviours on body mass index. Results from RESIDE', Preventive Medicine, vol. 53, no. 1-2, pp. 57-60. | Univariate analysis of how different variables affect BMI, one of them physical activity (PA) and another built environment (BE). No analysis for BE-PA |
| Christian, H, Knuiman, M, Bull, F, Timperio, A, Foster, S, Divitini, M, Middleton, N & Giles-Corti, B 2013, 'A new urban planning code's impact on walking: the residential environments project', American Journal Of Public Health, vol. 103, no. 7, pp. 1219-28. | Comparison of different neighbourhoods |
| D'Haese, S., Timperio, A., Veitch, J., Cardon, G., Van Dyck, D., & Salmon, J. (2013). Neighbourhood perceptions moderate the association between the family environment and children's objectively assessed physical activity. *Health and Place, 24*, 203-209. doi:10.1016/j.healthplace.2013.09.012 | Focus on children |
| Ding, D, Sallis, JF, Kerr, J, Lee, S & Rosenberg, DE 2011, 'Neighbourhood environment and physical activity among youth a review', American Journal Of Preventive Medicine, vol. 41, no. 4, pp. 442-55. | Systematic review |
| Durand, CP, Andalib, M, Dunton, GF, Wolch, J & Pentz, MA 2011, 'A systematic review of built environment factors related to physical activity and obesity risk: implications for smart growth urban planning', Obesity Reviews: An Official Journal Of The International Association For The Study Of Obesity, vol. 12, no. 5, pp. e173-e82. | Systematic review |
| Edwards, N. J., Giles-Corti, B., Larson, A., & Beesley, B. (2014). The effect of proximity on park and beach use and physical activity among rural adolescents. *Journal of Physical Activity and Health, 11*(5), 977-984. doi:10.1123/jpah.2011-0332 | Focus on adolescents |
| Foster, S, Giles-Corti, B & Knuiman, M 2010, 'Neighbourhood design and fear of crime: A social-ecological examination of the correlates of residents' fear in new suburban housing developments', Health and Place, vol. 16, no. 6, pp. 1156-65. | Outcome variable assessed is crime, not association presented for crime-PA |
| Gebel, K, Bauman, AE & Bull, FC 2010, 'Built environment: Walkability of neighbourhoods', in Evidence-Based Public Health: Effectiveness and Efficiency, DOI 10.1093/acprof:oso/9780199563623.003.019, via Scopus, <http://www.scopus.com/inward/record.url?eid=2-s2.0-84897466893&partnerID=40&md5=94c032235a8cd49c525d5a6e3a9a7edf>. | Book chapter with summary of evidence of BE-PA but none of the included studies meets the inclusion criteria |
| Giles-Corti, B, Kelty, SF, Zubrick, SR & Villanueva, KP 2009, 'Encouraging walking for transport and physical activity in children and adolescents: how important is the built environment?', Sports Medicine, vol. 39, no. 12, pp. 995-1009. | Focus on children |
| Giles-Corti, B., Wood, G., Pikora, T., Learnihan, V., Bulsara, M., Van Niel, K., . . . Villanueva, K. (2011). School site and the potential to walk to school: the impact of street connectivity and traffic exposure in school neighbourhoods. *Health & Place, 17*(2), 545-550. doi:10.1016/j.healthplace.2010.12.011 | Focus on children |
| Grasser, G, Van Dyck, D, Titze, S & Stronegger, W 2013, 'Objectively measured walkability and active transport and weight-related outcomes in adults: a systematic review', International Journal Of Public Health, vol. 58, no. 4, pp. 615-25. | One paper meeting inclusion criteria, Owen (2010), already found with search strategy |
| Hume, C, Jorna, M, Arundell, L, Saunders, J, Crawford, D & Salmon, J 2009, 'Are children's perceptions of neighbourhood social environments associated with their walking and physical activity?', Journal Of Science And Medicine In Sport / Sports Medicine Australia, vol. 12, no. 6, pp. 637-41. | Focus on social environment |
| Hunter, RF, Christian, H, Veitch, J, Astell-Burt, T, Hipp, JA & Schipperijn, J 2015, 'The impact of interventions to promote physical activity in urban green space: a systematic review and recommendations for future research', Social Science & Medicine (1982), vol. 124, pp. 246-56. | Systematic review |
| Hurni, A 2014, 'Investigating social and spatial dimensions of mobility with children and young people in Blacktown, Western Sydney', in Australasian Transport Research Forum, ATRF 2012 - Proceedings. | Focus on young people |
| Janssen, I 2014, 'Crime and perceptions of safety in the home neighbourhood are independently associated with physical activity among 11-15 year olds', Preventive Medicine, vol. 66, pp. 113-7. | Not Australian (Canadian) study and focus on adolescents |
| Koohsari, MJ, Sugiyama, T, Kaczynski, AT & Owen, N 2014, 'Associations of leisure-time sitting in cars with neighbourhood walkability', Journal Of Physical Activity & Health, vol. 11, no. 6, pp. 1129-32. | Outcome of interest is time sitting in cars, not physical activity |
| McCormack, GR & Shiell, A 2011, 'In search of causality: a systematic review of the relationship between the built environment and physical activity among adults', The International Journal Of Behavioural Nutrition And Physical Activity, vol. 8, pp. 125-. | Systematic review |
| McCormack, GR, Rock, M, Toohey, AM & Hignell, D 2010, 'Characteristics of urban parks associated with park use and physical activity: a review of qualitative research', Health & Place, vol. 16, no. 4, pp. 712-26. | Systematic review |
| McGrath, LJ, Hopkins, WG & Hinckson, EA 2015, 'Associations of Objectively Measured Built-Environment Attributes with Youth Moderate-Vigorous Physical Activity: A Systematic Review and Meta-Analysis', Sports Medicine (Auckland, N.Z.). | Focus on young people |
| Mees, P 2009, 'Density and transport mode choice in Australian, Canadian and US cities', in 32nd Australasian Transport Research Forum, ATRF 2009. | Not publicly available |
| Pont, K., Wadley, D., Ziviani, J., & Khan, A. (2013). The Influence of Urban Form and Family Decision Making on Children's Travel to School. *Journal of Urban Design, 18*(3), 363-382. doi:10.1080/13574809.2013.800452 | Focus on children |
| Rissel, C, Curac, N, Greenaway, M & Bauman, A 2012, 'Physical activity associated with public transport use-a review and modelling of potential benefits', International Journal Of Environmental Research And Public Health, vol. 9, no. 7, pp. 2454-78. (found in both searches, included in PA-Economic) | Public transport use as exposure variable, not physical activity |
| Sugiyama, T, Francis, J, Middleton, NJ, Owen, N & Giles-Corti, B 2010, 'Associations between recreational walking and attractiveness, size, and proximity of neighbourhood open spaces', American Journal Of Public Health, vol. 100, no. 9, pp. 1752-7. | No direct association, but through mediating variables |
| Sugiyama, T, Paquet, C, Howard, NJ, Coffee, NT, Taylor, AW, Adams, RJ & Daniel, M 2014, 'Public open spaces and walking for recreation: Moderation by attributes of pedestrian environments', Preventive Medicine, vol. 62, pp. 25-9. | No direct association, but through mediating variables |
| Timperio, A, Salmon, J, Ball, K, te Velde, SJ, Brug, J & Crawford, D 2012, 'Neighbourhood characteristics and TV viewing in youth: Nothing to do but watch TV?', Journal of Science and Medicine in Sport, vol. 15, no. 2, pp. 122-8. | Focus on young people and television viewing is the outcome of interest |
| Titze, S, Giles-Corti, B, Knuiman, MW, Pikora, TJ, Timperio, A, Bull, FC & van Niel, K 2010, 'Associations Between Intrapersonal and Neighbourhood Environmental Characteristics and Cycling for Transport and Recreation in Adults: Baseline Results From the RESIDE Study', Journal Of Physical Activity & Health, vol. 7, no. 4, pp. 423-31. | Assessment of the association of park visitation with PA outcome |
| Turrell, G, Haynes, M, Burton, NW, Giles-Corti, B, Oldenburg, B, Wilson, L-A, Giskes, K & Brown, WJ 2010, 'Neighbourhood disadvantage and physical activity: baseline results from the HABITAT multilevel longitudinal study', Annals of Epidemiology, vol. 20, no. 3, pp. 171-81. | Exposure is not built environment attributes |
| Turrell, G, Haynes, M, Wilson, L-A & Giles-Corti, B 2013, 'Can the built environment reduce health inequalities? A study of neighbourhood socioeconomic disadvantage and walking for transport', Health & Place, vol. 19, pp. 89-98. | Exposure is not built environment attributes |
| Turrell, G, Hewitt, B, Haynes, M, Nathan, A & Giles-Corti, B 2014, 'Change in walking for transport: a longitudinal study of the influence of neighbourhood disadvantage and individual-level socioeconomic position in mid-aged adults', The International Journal Of Behavioral Nutrition And Physical Activity, vol. 11, no. 1, pp. 151-. | Exposure is not built environment attributes |
| Van Dyck, D, Cerin, E, Conway, TL, De Bourdeaudhuij, I, Owen, N, Kerr, J, Cardon, G, Frank, LD, Saelens, BE & Sallis, JF 2012, 'Associations between perceived neighbourhood environmental attributes and adults' sedentary behaviour: Findings from the USA, Australia and Belgium', Social Science and Medicine, vol. 74, no. 9, pp. 1375-84. | Sitting outcome variable of interest and no country-specific estimated coefficients provided |
| Veitch, J, Ball, K, Crawford, D, Abbott, G & Salmon, J 2013, 'Is park visitation associated with leisure-time and transportation physical activity?', Preventive Medicine, vol. 57, no. 5, pp. 732-4. | Physical activity associated with park visitation, not with built environment attributes |
| Veitch, J, Timperio, A, Crawford, D, Abbott, G, Giles-Corti, B & Salmon, J 2011, 'Is the neighbourhood environment associated with sedentary behaviour outside of school hours among children?', Annals Of Behavioral Medicine: A Publication Of The Society Of Behavioral Medicine, vol. 41, no. 3, pp. 333-41. | Television viewing and computer game time outcomes of interest |
| Villanueva, K, Giles-Corti, B, Bulsara, M, McCormack, GR, Timperio, A, Middleton, N, Beesley, B & Trapp, G 2012, 'How far do children travel from their homes? Exploring children's activity spaces in their neighbourhood', Health and Place, vol. 18, no. 2, pp. 263-73. | Focus on children and outcome of interest is active spaces |
| Villanueva, K, Pereira, G, Knuiman, M, Bull, F, Wood, L, Christian, H, Foster, S, Boruff, BJ, Beesley, B, Hickey, S, Joyce, S, Nathan, A, Saarloos, D & Giles-Corti, B 2013, 'The impact of the built environment on health across the life course: Design of a cross-sectional data linkage study', BMJ Open, vol. 3, no. 1. | Presents study design protocol only |
| Wang, D, Brown, G & Liu, Y 2015, 'The physical and non-physical factors that influence perceived access to urban parks', Landscape and Urban Planning, vol. 133, pp. 53-66. | No measure of physical outcome |

Appendix D

Table 1 Summary of studies’ characteristics for built environment attributes-physical activity association for Australian adults

| **Authors (year)** | **Study characteristics**  Design  Location | **Sample recruitment**  Sample selection (SS)  Response Rate (RR) | **Project** | **Sample characteristics**  Number, age group, gender, others | **Geographic area** | **Physical activity (PA) outcome** | **Built environment variables (objective/perceived)** | **Self-selection adjustment** | **Confounding variables** |
| --- | --- | --- | --- | --- | --- | --- | --- | --- | --- |
| Astell-Burt, Feng (1) | Cross-sectional.  New South Wales | SS: Random form the Medicare Australia database (National provider of universal health insurance). *RR:* 18% | 45 and Up Study | n=203,883. Adults (≥45yrs.). 61.5 yrs.; 53.2% women; 32% household income less than $20,000 | Neighbourhood | (1) Walking and moderate vigorous physical activity (MVPA) for at least 10 minutes at least once per week; (2) Number of times of participation in MVPA. Self-reported. *Tool:* Active Australian Survey. Satisfactory levels of test-retest reliability. *Observation:* questionnaire does not specify where PA takes place. | **Objective:** Neighbourhood green space. *Measure:* Australian Meshblock classified as parkland. | No | **Socio-demographic:** age, gender, ethnicity, country of birth, body mass index, annual household income, education level, economic status, couple status, psychological distress, time spent outdoors and language other than English spoken at home. **Others:** measures of social interaction, neighbourhood affluence and neighbourhood remoteness. |
| Astell-Burt, Feng (2) | Cross-sectional. New South Wales | SS: Random form the Medicare Australia database (National provider of universal health insurance). *RR:* 18% | 45 and Up Study | n=203,883. Adults (≥45yrs.). 61.5 yrs.; 53.2% women; 32% household income less than $20,000 | Census Collection Districts (CCDs) (330 residents on average) and Statistical Local Areas (SLA) (32,000 residents on average) | Number of times of participation in MVPA in the last week. Self-reported. *Tool:* Active Australian Survey. Satisfactory levels of test-retest reliability and validity. *Observation:* questionnaire does not specify where PA takes place. | **Objective:** crime. *Measure:* New South Wales Bureau of Crime Statistic Research counts for same period of sample data. | No | **Socio-demographic:** age, gender, marital status, psychological distress, educational qualifications and annual household income and employment status. **Others:** neighbourhood affluence and geographical remoteness. |
| Christian, Bull (3) | Cross-sectional. Perth. Western Australia | SS: Respondents selected based on respondents plans to relocate into new developments. Random selection of household member. *RR:* 33.4% | RESIDE | Neighbourhood selection sample n=1,703. Adults (≥18yrs.) 39.9±11.9 yrs.; 59.5% women | Neighbourhood | Walking in the neighbourhood in a usual week for transport, leisure and in total. Total walking represent the addition of walking for transport and recreation. Outcomes for three thresholds (≥0mins. /wk., ≥60 mins. /wk. and 150 mins. /wk.). Self-reported. *Tool:* Neighbourhood PA Questionnaire (NPAQ). Acceptable reliability. *Observation*: questionnaire focus on PA in the neighbourhood. | **Objective:** Walkability index (WI) (net residential density, land use mix and street connectivity), and components of WI. *Measure:* Geographic Information Systems (GIS) | No | **Socio-demographic:** Gender, age, education level, marital status and presence of children at home for models assessing WI and components separately. **Built environment attributes:** density, connectivity and land use mix fitted simultaneously. |
| Cleland, Ball (4) | Cross-sectional. Victoria. | SS: Random selection of urban and rural areas in the bottom third of the Socio-Economic Index for Areas. Women randomly identify in each area. *RR:* 45% | READI | n=3765. Adults 34.6±8.2 yrs. All women. | Neighbourhood | Leisure time PA in the last week: >1 min/week, >120 min/week and >280 min/wk. Self-reported. *Tool:* International PA Questionnaire (long form). High reliability and acceptable validity. *Observation:* questionnaire does not specify where PA takes place. | **Perceived:** Environmental score. Cronbach’s alpha >0.63 for internal consistency. | No | **Socio-demographic**: age, number of children, country of birth, employment status, marital status. **Others:** areas of residence (urban/rural), smoking status, injury and illness and disability. |
| Duncan, Winkler (5) | Cross-sectional. Adelaide. South Australia. | SS: Resident of Census Collection Districts (CCDs) of the top and bottom walkability index (objective) randomly selected. Baseline data. *RR:* 11.5% (from other literature based on PLACE as not given in article) | PLACE^3^ | n=2506. Adults (20-64 yrs.). 44.3±12.3 mean yrs.; 64% women. Household income $31,200-$77,999 median. | Neighbourhood | (1) Minutes walking for transport per day; (2) Session per week. Self-reported. *Tool:* International PA Questionnaire (long form). Reporting for the previous 7 days.  *Observation:* questionnaire does not specify where PA takes place. | **Objective:** Land use mix. | No | **Socio-demographic:** age, gender, education, employment, household income, number of adults, presence of children in the household, and CCD-level median weekly household Income. |
| Foster, Knuiman (6) | Cross-sectional. Perth. Western Australia | SS: Cross-sectional data linkage. Stratified random sample for participants who completed the Western Australia Health and Wellbeing Surveillance System. *RR:* not given | Life course Built Environment and Health | n=3,487. Adults (25-65 yrs.); 47.4±10.5; 61.8% female | Neighbourhood | Self-reported times walked in the last week. Tool: Western Australian Health and Wellbeing Surveillance System. *Observation:* questionnaire does not specify where PA takes place. | **Objective:** Crime, residential density, street connectivity and local destinations. | No | **Socio-demographic:** age, sex, marital status, education and Index of Relative Socio-Economic Disadvantage (IRSD). **Built environment attributes:** density, street connectivity and local destinations. |
| Giles-Corti, Bull (7) | Quasi-experiment. Perth. Western Australia. Data after 12 (T1) and 36 months (T2) of relocation. Western Australia | SS: Participants selected based on plans to relocate into new developments. Random selection of household member. RR: T1: 36.4% and T2: 28.8%. | RESIDE | n=1437. Adults (>18yrs.). T1: 37.2±11.8 yrs. T2: 40.7±11.8 yrs.; T1: 52.3% women. T2: 37.6% women. | Neighbourhood | Change in mean minutes walked for transport and recreation in a usual week. Self-reported. *Tool:* Neighbourhood PA Questionnaire. High reliability and acceptable validity (not specified in article, from the literature using the same data set) *Observation*: questionnaire focus on PA in the neighbourhood. | **Objective:** Number out of seven of key transport-related walking destinations (range 0-7) that increased from T1 to T2. Number out of three of key recreation-related walking destinations (range 0-3) that increased from T1 to T2. **Perceived:** perceived access to mixed use and services, fewer cul de sacs, having footpaths on most streets; neighbourhood aesthetics shorter intersection distances, many alternative routes, slower traffic speeds, traffic slowing devices, accessibility of local parks or nature reserve, traffic safety, crime safety, infrastructure and safety for walking and local footpaths, hilly streets, the presence of major barrier | Yes, self-reported reasons for moving into a new neighbourhood as proxy of characteristics of importance in the built environment | **Socio-demographic:** baseline age, gender, education level, marital status, having children under 18 years at home, and baseline total minutes of recreational or transport related-walking. Transport related walking models included changes in work status, number of hours worked weekly and time to travel to work, while recreational walking models included changes in educational level. **Built environment attributes**: perceived changes in destination variables. |
| Heesch, Giles-Corti (8) | Cross-sectional.  Brisbane. Queensland. | SS: Participants randomly selected using a two stage cluster design (CCD and within CCD). CCD CCDs stratified by SES status. RR: 68.5% | HABITAT | n=10,233, Adults (40-65 yrs.). Baseline characteristics: 45-49 median yrs.; 55.6% women. 66.1% household income greater than $41,600 | Neighbourhood | (1) No cyclists: if reported recreational cycling less than monthly (last 12 months), (2) Recreational cyclist: if they reported recreational cycling at least monthly and no minutes of utilitarian cycling) (3) Utilitarian cyclists: if any minutes of transport cycling were reported, additional to having reported recreational cycling. Self-reported (1) Recreational cycling in the last 12 months: frequency (2) Transport cycling: time spent in the last week. *Observation:* questionnaire does not specify where PA takes place. | **Perceived:** traffic volume, neighbourhood aesthetics, crime, cul-de-sacs, recreational facilities and transport destinations, road traffic slowing devices, cul-de sacs (reverse coded), four-ways intersections and hilliness *Measure:* scales and items from the Neighbourhood Environment Walkability Scale (NEWS) with acceptable reliability. | No | **Socio-demographic:** age, sex and household composition. **Built environment attributes:** other included built environment attributes. **Others:** psychological disposition |
| Knuiman, Christian (9) | Quasi-experiment. Perth. Western Australia | SS: Participants selected based on plans to relocate into new developments. Random selection of household member. member. *RR:* 33.4% baseline | RESIDE | Baseline (n=1703), 1 year (n=1273), 3 years (1150) and 7 years (504). Adults (≥18yrs.). **Baseline characteristics:** 39.9±11.9 mean yrs.; 59.5% women. Household income $50,000-$69,000 median. | Neighbourhood | Walking for transport for more than 10 minutes in a week. Dichotomised. Self-reported duration of walking in a usual week. *Tool:* Neighbourhood Physical Activity Questionnaire. Acceptable reliability. *Observation*: questionnaire focus on PA in the neighbourhood. | **Objective:** Connectivity, residential density, land use mix, number of bus stops, number of train stations. **Perceived:** access to bus stops, access to railway stations, and access to services/convenience stores /public open spaces. *Measure*: GIS | Yes, three models presented, models 2 (logistic mixed model) and 3 (conditional logistic model) allowed for assessing the impact of self-selection. | **Socio-demographic variables:** Gender, age, education level, marital status, occupation, hours of work per week, annual income, number of adults living in the house, children living in the house and access to motor vehicle. **Built environment attributes:** other objective/perceived built environment attributes. |
| Koohsari, Kaczynski (10) | Cross sectional.  Melbourne. Victoria. | SS: Neighbourhoods selected purposively and individuals randomly within each neighbourhood type. RR: 35.3% |  | n=320. Adults (≥18yrs.). 44±15 mean yrs.; 56% females; 39% annual income more than $80,000 | Neighbourhood | (1) Walking to and within Public Open Space (POS); (2) Amount of walking in minutes in the last seven days. Self-reported. *Tool:* Questions adapted from the Physical Activity Questionnaire and the Neighbourhood Physical Activity Questionnaire. Reporting for the last 7 days. *Observation:* context specific questions of POS related walking. | **Objective:** Distance/Number/Area POS, neighbourhood local integration and control. *Measure:* GIS. **Perceived:** Attractiveness POS, neighbourhood facilities for walking/aesthetics/safety from crime/safety from traffic. *Measure:* selected sections of the Australian version of the Neighbourhood Environment Walkability Scale (NEWS-AUS) with validity proven elsewhere. | Yes, the variable "Closeness to POS" used to control for self-selection (reported). | **Socio-demographic:** age, gender, employment status, income, education level, dog ownership and children in the household. **Built environment attributes:** perceptual qualities of the neighbourhood: aesthetics, safety from traffic, safety from crime, and the availability of facilities for walking |
| Koohsari, Karakiewicz (11) | Cross sectional. Melbourne. Victoria. | SS: Neighbourhoods selected purposively and individuals randomly within each neighbourhood type. RR: 35.3% |  | n=320. Adults (≥18yrs.). 44±15 mean yrs.; 56% females; 39% annual income more than $80,000 | Neighbourhood | (1) Some walking to POS, (2) Some walking within POS. *Tool:* Self-reported. International Physical Activity Questionnaire (long form). Reporting for the previous 7 days. *Observation:* context specific questions of POS related walking. | **Objective:** distance/number/area/integration POS. *Measure*: GIS. | Yes, the variable "Closeness to POS" used to control for self-selection (reported). | **Socio-demographic:** age, gender, employment status, income, education level, dog ownership and children in the household. **Built environment attributes:** neighbourhood quality (perceived), and POS attractiveness (perceived) |
| Koohsari, Sugiyama (12) | Cross-sectional. Adelaide. South Australia. | SS: Resident of CCDs of the top and bottom walkability index (objective) randomly selected. RR: 11.5% | PLACE | n=2506. Adults (20-64 yrs.). 44.3±12.3 mean yrs.; 64% women. Household income $31,200-$77,999 median. | Neighbourhood | Days of walking for transport. Self-reported. *Tool:* International Physical Activity Questionnaire (long form). Reporting for the previous 7 days. Adequate reliability and validity. *Observation:* questionnaire does not specify where PA takes place. | **Objective:** Street connectivity. *Measure:* GIS. **Perceived:** number of utilitarian destinations. | No | **Socio-demographic**: age, gender, education attainment, work status, marital status, having children in the households, annual household income, car ownership, and neighbourhood economic status. **Built environment attributes**: all variables included. |
| Learnihan, Van Niel (13) | Cross sectional. Perth. Western Australia | SS: Respondents selected based on respondents plans to relocate into new developments. Random selection of household member. *RR:* 33.4% | RESIDE | n=1753. Adults (>18yrs). 38 median yrs.; 60% women. | Neighbourhood | (1) Doing any walking in the neighbourhood for transport or leisure (yes/no), (3) Meeting 150 min/week walking general and transport. *Tool:* Neighbourhood Physical Activity Questionnaire. *Observation*: questionnaire focus on PA in the neighbourhood. | **Objective:** walkability index composed of connectivity, residential land use and land use mix. *Measure*: GIS | No | **Socio-demographic**: age, gender, education and household income. |
| McCormack, Shiell (14) | Cross sectional. Perth. Western Australia | SS: Respondents selected based on respondents plans to relocate into new developments. Random selection of household member. *RR:* 33.4% | RESIDE | n=1,681. Adults (≥18yrs.) 40.16±11.96 yrs.; 58.7% women | Neighbourhood | (1) Participation in walking vs no participation (binary outcome), (2) in those who walked, walking minutes (non-randomly self-selected) for leisure, for transport and total walking. Self-reported duration of walking in a usual week. *Tool:* NPAQ. Acceptable reliability. *Observation*: questionnaire focus on PA in the neighbourhood. | **Objective:** walkability index and sidewalk length. *Measure:* GIS. **Perceived:** access to recreation/schools/services, streets pedestrian/cycle friendly and house affordability/variety | Yes, self-reported reasons for moving into a new neighbourhood as proxy of characteristics of importance in the built environment | **Socio-demographic:** gender, age, education. **Attitudes**: attitudes towards walking and neighbourhood preference. **Neighbourhood attitude. Built environment attributes:** walkability, sidewalks |
| McKibbin (15) | Cross-sectional Greater Sydney region. New South Wales | Not reported |  | Not reported | Greater Sydney | Non-car mode share. Not clear data source and method of measurement. | **Objective.** Density (population/jobs), diversity, design, destination accessibility (walking/by car and distance to transit. | No | **Socio-demographics:** income, car ownership and destination of work trips. **Built environment attributes:** all built environment attributes fitted simultaneously. |
| Owen, De Bourdeaudhuij (16) | Cross-sectional. Adelaide. South Australia. | SS: Resident of CCDs of the top and bottom walkability index (objective) randomly selected. RR: 11.5% | PLACE | n=2194; adults (20-65 yrs.); 45.5±11.8; 56% women; 51.2% income >$41,600 annually. | Neighbourhood | Bicycle use at least once per week for more than 10 minutes. Self-reported. *Tool:* Questions from the International Physical Activity Questionnaire (long form). Reliability and validity tested. *Observation:* questionnaire does not specify where PA takes place. | **Objective:** walkability index | No | **Socio-demographic:**  age, gender, education, working status, and area level SES |
| Shimura, Sugiyama (17) | Longitudinal. Adelaide. South Australia. | SS: Resident of CCDs of the top and bottom walkability index (objective) randomly selected. RR: 11.5%Follow up: 41.4% of those who completed baseline. | PLACE | n=504; adults (20-65 yrs.); 57 (median age); 54% women; 53.8% income>$41,600 per year | Neighbourhood | Changes in time spent walking between baseline and follow up(transport and recreation separately). Self-reported. *Tool:* International PA questionnaire (long form). *Observation:* questionnaire does not specify where PA takes place. | **Objective:** walkability index. *Measure:* GIS | No | **Socio-demographic:** age, gender, work status, household income, and BMI. **Other:** time spent walking for transport and recreation at baseline. |
| Sugiyama, Cerin (18) | Cross-sectional. Adelaide. South Australia. | SS: Resident of CCDs of the top and bottom walkability index (objective) randomly selected. RR: 11.5% | PLACE | n=2650. Adults (18-66 yrs.); 44 (12.3); 64% women | Neighbourhood | Minutes of walking for recreation in the last seven days. Self-reported. *Tool:* International Physical Activity Questionnaire-Long Version. Good test retest reliability and fair-to-moderate criterion validity. *Observation:* questionnaire does not specify where PA takes place. | **Perceived:** aesthetics. *Measure:* NEWS | No | **Socio-demographic:**  age, gender, marital status, educational attainment, work status, neighbourhood and SES. **Built environment attributes:** perceived environmental characteristics (residential density score, land use mix, connectivity, infrastructure and safety, safety from traffic, safety from crime, few cul-de-sacs, no major barriers and proximity to parks). |
| Sugiyama, Giles-Corti (19) | Longitudinal. Perth. Adelaide. South Australia | SS: Resident of CCDs of the top and bottom walkability index (objective) randomly selected. RR: 11.5%Follow up: 41.4% of those who completed baseline. | PLACE | n=681; 48.6±10.2; adults; 60.9% women; 53.5% income >$41,600 | Neighbourhood | (1) Initiated recreational walking from baseline to after 12 months, (2) Maintained recreational walking from between baseline and 12 months after. Question of walking frequency for more than 10 minutes in the last 7 days. *Tool:* International PA questionnaire (long form). *Observation:* questionnaire does not specify where PA takes place. | **Objective:** POS area/size/number. *Measure:* GIS. **Perceived:** POS presence/quality/proximity | No | **Socio-demographic:** gender, age, work status, marital status, walking for transport and TV viewing time. **Others:** the index of facing the sea (only for GIS measure) |
| Sugiyama, Leslie (20) | Cross-sectional. Adelaide, South Australia. | SS: Resident of CCDs of the top and bottom walkability index (objective) randomly selected. RR: 11.5% | PLACE | n=2194; adults (20-65 yrs.); 45.5±11.8; 56% women; 51.2% income >$41,600 annually. | Neighbourhood | Number of days that participants used streets near home for moderate to vigorous PA for recreation or exercise in the last month. Variable dichotomised at the median. Self-reported number of days in the past month of MVPA. *Observation:* questions specified PA near home. | **Perceived:** neighbourhood attractiveness, street connectivity and presence of sidewalks. *Measure:* NEWS | No | **Socio-demographic:** age, gender, educational attainment, work status and annual household income |
| Titze, Giles-Corti (21) | Cross sectional. Perth. Western Australia | SS: Respondents selected based on respondents plans to relocate into new developments. Random selection of household member. *RR:* 33.4% | RESIDE | n=1813; adults; women (58.5%); 40±(11.9); 40% completed secondary education; 93% reported access to moto vehicle. | Neighbourhood | Cycling for transport and recreation within and without the neighbourhood in the last week. *Observation:* information on cycling with and without the neighbourhood was combined for analyses. | **Perceived-Cycling for recreation:** Presence of many alternative routes. **Cycling for transport:** neighbourhood surrounding leafy and attractive, cycle/walking path available/presence of many traffic slowing devices/presence of many 4-way intersections/presence of many alternative routes. *Measure*: NEWS. | No | **Sociodemographic:** age, gender, education. **Built environment attributes:** all built environment attributes simultaneously fitted in regression. **Others:** car availability, attitudes towards cycling and perceived behavioural control for cycling. |
| Villanueva, Knuiman (22) | Cross sectional. Perth. Western Australia | SS: Cross-sectional data linkage. Stratified random sample for participants who completed the Western Australia Health and Wellbeing Surveillance System. *RR:* not given | Life Course Built Environment and Health | Final sample used for analyses is not clearly stated. | Neighborhood | Self-reported times walked in the last week. Tool: Western Australian Health and Wellbeing Surveillance System. *Observation:* questionnaire does not specify where PA takes place. | **Objective:** walkability index. *Measure:* GIS | No | **Socio-demographic:** gender, age, education, socio-economic status |
| Wilson, Giles-Corti (23) | Cross-sectional.  Brisbane. Queensland. | SS: Participants randomly selected using a two stage cluster design (CCD and within CCD). CCD CCDs stratified by SES status. RR: 68.5% | HABITAT | Baseline (n=10,286). Adults (40-65 yrs.). **Baseline characteristics:** 45-49 median yrs.; 55.7% women. Household income $41,600-51,999 median. |  | Total minutes walked in the last week (thresholds: 30mins/wk., ≥30-<90, ≥90-<150, ≥150-<300 and , ≥300). Total walking represents time spent walking for recreation, for exercise, or to get to and from places .*Tool:* Active Australian survey. Acceptable reliability and validity. *Observation:* questionnaire does not specify where PA takes place. | **Objective:** connectivity, density (residential), hilliness, tree coverage, bikeways, streetlights, river to coast (distance), public transport (distance), shops (distance) and parks (distance). *Measure:* GIS | No | **Socio-demographic**: Index of relative socioeconomic disadvantage, age, sex, household type, education level and household income. |

**1**. RESIDendial Environments project. **2.** Resilience for Eating and Activity Despite Inequality. **3.** Physical Activity in Localities and Community Environment. **4.** **H**ow **A**reas in **B**risbane **I**nfluence heal**T**h **A**nd ac**T**ivity

**References**

1. Astell-Burt T, Feng X, Kolt GS. Green space is associated with walking and moderate-to-vigorous physical activity (MVPA) in middle-to-older-aged adults: findings from 203 883 Australians in the 45 and Up Study. British Journal Of Sports Medicine. 2014;48(5):404-6.

2. Astell-Burt T, Feng X, Kolt GS. Identification of the impact of crime on physical activity depends upon neighbourhood scale: Multilevel evidence from 203,883 Australians. Health & Place. 2015;31:120-3.

3. Christian HE, Bull FC, Middleton NJ, Knuiman MW, Divitini ML, Hooper P, et al. How important is the land use mix measure in understanding walking behaviour? Results from the RESIDE study. The International Journal Of Behavioral Nutrition And Physical Activity. 2011;8:55-.

4. Cleland VJ, Ball K, Crawford D. Is a perceived supportive physical environment important for self-reported leisure time physical activity among socioeconomically disadvantaged women with poor psychosocial characteristics? An observational study. BMC Public Health. 2013;13:280-.

5. Duncan MJ, Winkler E, Sugiyama T, Cerin E, duToit L, Leslie E, et al. Relationships of land use mix with walking for transport: do land uses and geographical scale matter? Journal Of Urban Health: Bulletin Of The New York Academy Of Medicine. 2010;87(5):782-95.

6. Foster S, Knuiman M, Villanueva K, Wood L, Christian H, Giles-Corti B. Does walkable neighbourhood design influence the association between objective crime and walking? The International Journal Of Behavioral Nutrition And Physical Activity. 2014;11(1):100-.

7. Giles-Corti B, Bull F, Knuiman M, McCormack G, Van Niel K, Timperio A, et al. The influence of urban design on neighbourhood walking following residential relocation: longitudinal results from the RESIDE study. Social Science & Medicine (1982). 2013;77:20-30.

8. Heesch KC, Giles-Corti B, Turrell G. Cycling for transport and recreation: associations with socio-economic position, environmental perceptions, and psychological disposition. Prev Med. 2014;63:29-35.

9. Knuiman MW, Christian HE, Divitini ML, Foster SA, Bull FC, Badland HM, et al. A longitudinal analysis of the influence of the neighborhood built environment on walking for transportation: the RESIDE study. American Journal Of Epidemiology. 2014;180(5):453-61.

10. Koohsari MJ, Kaczynski AT, Giles-Corti B, Karakiewicz JA. Effects of access to public open spaces on walking: Is proximity enough? Landscape and Urban Planning. 2013;117:92-9.

11. Koohsari MJ, Karakiewicz JA, Kaczynski AT. Public Open Space and Walking: The Role of Proximity, Perceptual Qualities of the Surrounding Built Environment, and Street Configuration. Environment and Behavior. 2013;45(6):706-36.

12. Koohsari MJ, Sugiyama T, Lamb KE, Villanueva K, Owen N. Street connectivity and walking for transport: role of neighborhood destinations. Preventive Medicine. 2014;66:118-22.

13. Learnihan V, Van Niel KP, Giles-Corti B, Knuiman M. Effect of Scale on the Links between Walking and Urban Design. Geographical Research. 2011;49(2):183-91.

14. McCormack GR, Shiell A, Giles-Corti B, Begg S, Veerman JL, Geelhoed E, et al. The association between sidewalk length and walking for different purposes in established neighborhoods. International Journal of Behavioral Nutrition and Physical Activity. 2012;9.

15. McKibbin M, editor The influence of the built environment on mode choice - Evidence from the journey to work in Sydney. ATRF 2011 - 34th Australasian Transport Research Forum; 2014.

16. Owen N, De Bourdeaudhuij I, Sugiyama T, Leslie E, Cerin E, Van Van Dyck D, et al. Bicycle use for transport in an Australian and a Belgian city: associations with built-environment attributes. Journal Of Urban Health: Bulletin Of The New York Academy Of Medicine. 2010;87(2):189-98.

17. Shimura H, Sugiyama T, Winkler E, Owen N. High neighborhood walkability mitigates declines in middle-to-older aged adults' walking for transport. Journal Of Physical Activity & Health. 2012;9(7):1004-8.

18. Sugiyama T, Cerin E, Owen N, Oyeyemi AL, Conway TL, Van Dyck D, et al. Perceived neighbourhood environmental attributes associated with adults[U+05F3] recreational walking: IPEN Adult study in 12 countries. Health and Place. 2014;28:22-30.

19. Sugiyama T, Giles-Corti B, Summers J, du Toit L, Leslie E, Owen N. Initiating and maintaining recreational walking: a longitudinal study on the influence of neighborhood green space. Preventive Medicine. 2013;57(3):178-82.

20. Sugiyama T, Leslie E, Giles-Corti B, Owen N. Physical activity for recreation or exercise on neighbourhood streets: associations with perceived environmental attributes. Health & Place. 2009;15(4):1058-63.

21. Titze S, Giles-Corti B, Knuiman MW, Pikora TJ, Timperio A, Bull FC, et al. Associations Between Intrapersonal and Neighborhood Environmental Characteristics and Cycling for Transport and Recreation in Adults: Baseline Results From the RESIDE Study. Journal of Physical Activity & Health. 2010;7(4):423-31.

22. Villanueva K, Knuiman M, Nathan A, Giles-Corti B, Christian H, Foster S, et al. The impact of neighborhood walkability on walking: does it differ across adult life stage and does neighborhood buffer size matter? Health & Place. 2014;25:43-6.

23. Wilson L-AM, Giles-Corti B, Burton NW, Giskes K, Haynes M, Turrell G. The association between objectively measured neighborhood features and walking in middle-aged adults. American Journal Of Health Promotion: AJHP. 2011;25(4):e12-e21.

**Appendix E Table 1 Summary of findings**

| **Indicators** | **Domain** | | | | | | | | **Objective BE** | | **Perceived BE** | |
| --- | --- | --- | --- | --- | --- | --- | --- | --- | --- | --- | --- | --- |
|  | **Transport** | | | **Leisure** | | | **Total** | | **All studies** | **Good and fair quality** | **All studies** | **Good and fair quality** |
| **Density** | | | | | | | | | **3/9 (33%) [4]** | **1/5 (20%) [2]** |  |  |
| Population | **0 (1)^1^/0 (2)^2^/ + (3)**** | |  | **0 (1) ^1^** | |  | **0 (1) ^1^/+(4)***/ 0 (5)^3^/ 0 (5) ^3^** |  | 2/8 [4] | 1/5 [2] |  |  |
| Jobs density | **+ (3)**** | |  |  | |  |  |  | 1/1 [1] |  |  |  |
| **Diversity** | | | | | | | | | **4/6 (67%) [3]** | **2/4 (50%) [1]** |  |  |
| Land use mix and non-residential zone | **+ (1)** ^1^/ + (6)*/**  **+ (2) **^2^/ + (3)**** | |  | **0 (1) ^1^** |  | | **0 (1) ^1^** |  | 4/6 [3] | 2/4 [1] |  |  |
| **Design** | | | | | | | | | **8/29 (28%) [6]** | **6/24 (25%) [4]** | **16/32 (50%) [3]** | **11/27 (40%) [3]** |
| **Street network** | | | | | | | | | **4/11 (36%) [5]** | **4/8 [50%] [4]** | **6/7 (86%) [3]** | **3/4 (75%) [2]** |
| Connectivity/ Space syntax measures: local integration and control integration/Cul de sacs | **+ (1)** ^1^/ + (7) ***^6^/**  **+ (2)** ^2^** | 0 (8) | | **0 (1) ^1^/ - (9) ***^7^/ - (9) ^7^***** | + (10) **^8^/ + (10) ** ^9^/ + (8)** | | **0 (1) ^1^/ + (4) *** / 0 (5) ^3^/ 0 (5) ^3^** |  | 4/10 [4] | 4/8 [4] | 3/4 [2] | 3/4 [2] |
| Traffic slowing devices, pedestrian crossings, road traffic volume and busy roads/3 or more ways intersections |  | + (11)**/ + (11)** | |  |  | |  |  |  |  | 2/2 [1] |  |
| Functionality/Route options |  |  | |  | + (11)** | |  |  |  |  | 1/1 [1] |  |
| Street density | **0 (3)** |  | |  |  | |  |  | 0/1 [1] |  |  |  |
| **Transport infrastructure** | | | | | | | | | **2/5 (40%) [2]** | **2/5 (40%) [2]** | **6/11 (40%) [4]** | **4/9 (44%) [3]** |
| Sidewalks/bikeways | **0 (12)^10^** | + (11)** | | **0 (12) ^10^** | 0 (10) ^8^ / 0 (10) ^9^/ 0 (9)^7^ | | **+ (12) **^10^/ +(4)***** |  | 2/4 [2] | 2/4 [2] | 1/4 [3] | 0/3 [2] |
| Street lights/ Aesthetics/Attractiveness |  | 0 (8)/ + (11)** | |  | + (8)**/ + (9) *^7^/ 0 (13)/  + (10) / + (10) | | **0 (4)** |  | 0/1 [1] | 0/1 [1] | 5/7 [4] | 4/6 [3] |
| **Green and recreational space** | | | | | | | | | **2/13 (15%) [2]** | **0/11 (0%) [1]** | **4/14 (29%) [2]** | **4/14 (29%) [2]** |
| Green space/POS/sports facilities-**area/presence** |  |  | | **0 (9) ^7^/0 (15)^13^/ 0 (15)^14^** | 0 (14)^11^/+ (14)^12^ /0 (14) ^11^ /+ (14) **^12^/0 (14)^11^  / + (14) **^12^ | | **+ (16)**/ + (16)**** |  | 2/5 [2] | 0/3 [1] | 3/6 [1] | 3/6 [1] |
| Green space/POS/sports facilities -**distance** |  |  | | **0 (9) ^7^/0 (15)^13^/ 0 (15)^14^** | 0 (9) **^7^**/ 0 (14)^11^/  + (14) **^12^ | |  |  | 0/3 [1] | 0/3 [1] | 1/3 [2] | 1/3 [2] |
| Green space/POS/sports facilities -**number** |  |  | | **0 (9) ^7^/0 (15)^13^/ 0 (15)^14^** | 0 (14)^11^/ 0 (14)^12^ | |  |  | 0/3 [1] | 0/3 [1] | 0/2 [1] | 0/2 [1] |
| Space syntax measure: integration public open space |  |  | | **0 (15)^13^/ 0 (15)^14^** |  | |  |  | 0/2 [1] | 0/2 [1] |  |  |
| Green spaces quality/attractiveness/aesthetics/maintenance |  |  | |  | 0 (14)^11^/ 0 (14)^12^/0 (9)**^7^** | |  |  |  |  | 0/3 [2] | 0/3 [2] |
| **Destinations** | | | | | | | | | **7/10 (70%) [4]** | **3/6 (50%) [2]** | **10/14 (71%) [3]** | **10/14 (71%) [3]** |
| **Transport related** | | | | | | | | | **6/8 (75%) [4]** | **2/4 (50%) [2]** | **5/6 (83%) [3]** | **5/6 (83%) [3]** |
| Transport related destinations | **+ (17)**^15^/ 0 (2)^2^ / 0 (2)** | + (17)** **^15^**/ 0 (8)/ + (2)** / + (2)** / + (7) ***^6^ | |  | + (8)** | | **+ (5) **^3^/ + (5) **^3^/ + (4)***** |  | 4/6 [3] | 2/4 [2] | 5/6 [3] | 5/6 [3] |
| Jobs accessibility by public transport | **+ (3)**** |  | |  |  | |  |  | 1/1 [1] |  |  |  |
| Jobs accessibility by car | **- (3)**** |  | |  |  | |  |  | 1/1 [1] |  |  |  |
| **Recreation related** | | | | | | | | | **1/2 (50%) [2]** | **1/2 (50%) [2]** | **5/7 (71%) [3]** | **5/7 (71%) [3]** |
| Recreational related destinations distance (places of interest, recreation, parks) |  | + (8) ****** | | **+ (17)* ^15^** | + (17)** **^15^**/ + (8)**/  0 (10)^8^/+ (10)**^9^/ 0 (10)^8^/+ (10)**^9^ | | **0 (4)** |  | 1/2 [2] | 1/2 [2] | 5/7 [3] | 5/7 [3] |
| **Distance to transit** | | | | | | | | | **4/5 (80%) [3]** | **3/4 (75%) [2]** | **1/2 (50%) [1]** | **1/2 (50%) [1]** |
| Bus stops/train stations | **+ (2)**^2^/ + (2)** ^2^/ + (2)**^2^/ + (3)**** | 0 (2)^2^/ + (2)**^2^ | |  |  | | **0 (4)** |  | 4/5 [3] | 3/4 [2] | 1/2 [1] | 1/2 [1] |
| **Safety** | | | | | | | | | **2/6 (33%) [2]** | **0/0 (0%)** | **3/9 (33%) [3]** | **3/9 (33%) [3]** |
| Safety from crime |  | 0 (8) | |  | + (9) **^7^/ + (8)/ 0 (10)/ 0 (10) | | **+ (18)**^16^/ + (18)** ^16^/ - (5)**^4^/ -(5) **^4^/ - (5) **^4^/ - (5) ** ^4^** |  | 2/6 [2] | 0/0 | 2/5 [3] | 2/5 [3] |
| Traffic safety |  |  | |  | + (9) ***^7^/ 0 (10)/0 (10) | |  |  |  |  | 1/4 [2] | 1/4 [2] |
| **Aggregated neighbourhood measures** | | | | | | | | | **14/19 (74%) [3]** | **8/15 53% [2]** | **1/1 (100%) [1]** | **1/1 (100%) [1]** |
| Walkability index | **+ (1)**/ + (19)**/**  **+ (19)**/ + (19)** /**  **+ (12)**^10^/ + (20)**/+ (23)***** |  | | **0 (1)/ 0 (19)/ + (19)**/**  **+ (19)**/ 0 (20)/0 (12) ^10^** | + (21)** | | **+ (1)**/**  **0 (12) ^10^/ + (22)***^17^ /**  **+ (22)*** ^17^  /+ (22)*** ^17^ /+ (22)*** ^17^ /** |  | 12/19 [3] | 8/15 [2] | 1/1 | 1/1 |

Notes *, **, *** indicates statistical significance at 10%, 5% and 1% level. Regular font=subjective BE; bold font: objective BE measure; **1.** Model 2 Christian, Bull (1). **2**. Model 3 Knuiman, Christian (2). **3.** Model 1 Foster, Knuiman (5). **4.** Models 2 Foster, Knuiman (5). **5.** Area-corrected revised LUM score Duncan, Winkler (6). **6.** Model 2 (7). **7.** Model for “Some walking to/within POS” Koohsari, Karakiewicz (9). **8.** Model 1 (10).**9.** Model 2 (10) **10**. Model 2 (12). **11.** Model for initiation of recreational walking Sugiyama, Giles-Corti (14). **12.** Model for maintenance of recreational walking Sugiyama, Giles-Corti (14). **13** Model for some walking to POS (15). **14.** Model for walking within POS (15)). **15.** Model 4 + self-selection Giles-Corti, Bull (17). **16**. Model 1 Astell-Burt, Feng (18). **17.** Model “All adults” Villanueva, Knuiman (22)

**References**

1. Christian H, Bull F, Middleton NJ, Knuiman MW, Divitini ML, Hooper P, et al. How important is the land use mix measure in understanding walking behaviour? Results from the RESIDE study. The International Journal Of Behavioral Nutrition And Physical Activity. 2011;8:55-.

2. Knuiman MW, Christian HE, Divitini ML, Foster SA, Bull FC, Badland HM, et al. A longitudinal analysis of the influence of the neighborhood built environment on walking for transportation: the RESIDE study. American Journal Of Epidemiology. 2014;180(5):453-61.

3. McKibbin M, editor The influence of the built environment on mode choice - Evidence from the journey to work in Sydney. ATRF 2011 - 34th Australasian Transport Research Forum; 2014.

4. Wilson L-AM, Giles-Corti B, Burton NW, Giskes K, Haynes M, Turrell G. The association between objectively measured neighborhood features and walking in middle-aged adults. American Journal Of Health Promotion: AJHP. 2011;25(4):e12-e21.

5. Foster S, Knuiman M, Villanueva K, Wood L, Christian H, Giles-Corti B. Does walkable neighbourhood design influence the association between objective crime and walking? The International Journal Of Behavioral Nutrition And Physical Activity. 2014;11(1):100-.

6. Duncan MJ, Winkler E, Sugiyama T, Cerin E, duToit L, Leslie E, et al. Relationships of land use mix with walking for transport: do land uses and geographical scale matter? Journal Of Urban Health: Bulletin Of The New York Academy Of Medicine. 2010;87(5):782-95.

7. Koohsari MJ, Sugiyama T, Lamb KE, Villanueva K, Owen N. Street connectivity and walking for transport: role of neighborhood destinations. Preventive Medicine. 2014;66:118-22.

8. Heesch KC, Giles-Corti B, Turrell G. Cycling for transport and recreation: associations with socio-economic position, environmental perceptions, and psychological disposition. Prev Med. 2014;63:29-35.

9. Koohsari MJ, Karakiewicz JA, Kaczynski AT. Public Open Space and Walking: The Role of Proximity, Perceptual Qualities of the Surrounding Built Environment, and Street Configuration. Environment and Behavior. 2013;45(6):706-36.

10. Sugiyama T, Leslie E, Giles-Corti B, Owen N. Physical activity for recreation or exercise on neighbourhood streets: associations with perceived environmental attributes. Health & Place. 2009;15(4):1058-63.

11. Titze S, Giles-Corti B, Knuiman MW, Pikora TJ, Timperio A, Bull FC, et al. Associations Between Intrapersonal and Neighborhood Environmental Characteristics and Cycling for Transport and Recreation in Adults: Baseline Results From the RESIDE Study. Journal of Physical Activity & Health. 2010;7(4):423-31.

12. McCormack GR, Shiell A, Giles-Corti B, Begg S, Veerman JL, Geelhoed E, et al. The association between sidewalk length and walking for different purposes in established neighborhoods. International Journal of Behavioral Nutrition and Physical Activity. 2012;9.

13. Sugiyama T, Cerin E, Owen N, Oyeyemi AL, Conway TL, Van Dyck D, et al. Perceived neighbourhood environmental attributes associated with adults[U+05F3] recreational walking: IPEN Adult study in 12 countries. Health and Place. 2014;28:22-30.

14. Sugiyama T, Giles-Corti B, Summers J, du Toit L, Leslie E, Owen N. Initiating and maintaining recreational walking: a longitudinal study on the influence of neighborhood green space. Preventive Medicine. 2013;57(3):178-82.

15. Koohsari MJ, Kaczynski AT, Giles-Corti B, Karakiewicz JA. Effects of access to public open spaces on walking: Is proximity enough? Landscape and Urban Planning. 2013;117:92-9.

16. Astell-Burt T, Feng X, Kolt GS. Green space is associated with walking and moderate-to-vigorous physical activity (MVPA) in middle-to-older-aged adults: findings from 203 883 Australians in the 45 and Up Study. British Journal Of Sports Medicine. 2014;48(5):404-6.

17. Giles-Corti B, Bull F, Knuiman M, McCormack G, Van Niel K, Timperio A, et al. The influence of urban design on neighbourhood walking following residential relocation: longitudinal results from the RESIDE study. Social Science & Medicine (1982). 2013;77:20-30.

18. Astell-Burt T, Feng X, Kolt GS. Identification of the impact of crime on physical activity depends upon neighbourhood scale: Multilevel evidence from 203,883 Australians. Health & Place. 2015;31:120-3.

19. Learnihan V, Van Niel KP, Giles-Corti B, Knuiman M. Effect of Scale on the Links between Walking and Urban Design. Geographical Research. 2011;49(2):183-91.

20. Shimura H, Sugiyama T, Winkler E, Owen N. High neighborhood walkability mitigates declines in middle-to-older aged adults' walking for transport. Journal Of Physical Activity & Health. 2012;9(7):1004-8.

21. Cleland VJ, Ball K, Crawford D. Is a perceived supportive physical environment important for self-reported leisure time physical activity among socioeconomically disadvantaged women with poor psychosocial characteristics? An observational study. BMC Public Health. 2013;13:280-.

22. Villanueva K, Knuiman M, Nathan A, Giles-Corti B, Christian H, Foster S, et al. The impact of neighborhood walkability on walking: does it differ across adult life stage and does neighborhood buffer size matter? Health & Place. 2014;25:43-6.

23. Owen N, De Bourdeaudhuij I, Sugiyama T, Leslie E, Cerin E, Van Van Dyck D, et al. Bicycle use for transport in an Australian and a Belgian city: associations with built-environment attributes. Journal Of Urban Health: Bulletin Of The New York Academy Of Medicine. 2010;87(2):189-98.

**Appendix F**

Table 1 Quality assessment cross sectional studies

| Ref. | **Publication** | **Response rate** 2=>60%, 1=41-60% 0=<40% or not known | **Representativness**  (whether repondents have similar characteristics as population represented)  2=if stated that representative or comparison with population provided 1=if some minor limitations  0=not met or not known | **Outcome measures**  2=validated questionnare  1=self-reported  0=not met or not known | **Confounding**  2=on individual and neighbourhood built environment attributes  1=on individual level  0=not met or not known  0.5 additional if control for self-selction | **Global rating** |
| --- | --- | --- | --- | --- | --- | --- |
| **1** | **Astell-Burt et al. (2014)** | 0 | 0 (not known) | 2 | 1 | 3 (Poor) |
| **2** | **Astell-Burt et al. (2015)** | 0 | 0 (not known) | 2 | 1 | 3 (Poor) |
| **3** | **Christian et al. (2011)** | 0 | 0 (not met) | 2 | 2 | 4 (Fair) |
| **4** | **Cleland et al. (2013)** | 1 | 0 (not known) | 2 | 2**^1^** | 4 (Fair) |
| **5** | **Duncan et al. (2010)** | 0 | 0 (not known) | 2 | 1 | 3 (Poor) |
| **6** | **Foster et al. (2014)** | 0 (not known) | 0 (not known) | 1 | 2 | 3 (Poor) |
| **7** | **Heesch et al. (2014)** | 2 | 2 | 1 | 2 | 7 (Good) |
| **8** | **Koohsari et al. (2013)** | 0 | 0 (not known) | 2 | 2.5 | 4.5 (Fair) |
| **9** | **Koohsari (Karakiewicz) et al. (2013)** | 0 | 0 (not known) | 2 | 2.5 | 4.5 (Fair) |
| **10** | **Koohsari et al. (2014)** | 0 | 0 (not known) | 2 | 2 | 4 (Fair) |
| **11** | **Learnihan et al. (2011)** | 0 | 0 (not met) | 2 | 2**^2^** | 4 (Fair) |
| **12** | **McCormack et al. (2012)** | 0 | 0 (not met) | 2 | 2.5 | 4.5 (Fair) |
| **13** | **McKibbin (2014)** | 0 (not known) | 0 (not known) | 0 (not known) | 2 | 2 (Poor) |
| **14** | **Owen et al. (2010)** | 0 | 0 (not known) | 2 | 2 | 4 (Fair) |
| **15** | **Sugiyama et al. (2014)** | 0 | 0 (not known) | 2 | 2 | 4 (Fair) |
| **16** | **Sugiyama et al. (2009)** | 0 | 0 (not known) | 2 | 2 | 4 (Fair) |
| **17** | **Titze et al. (2010)** | 0 | 0 (not met) | 1 | 2 | 3 (Poor) |
| **18** | **Villanueva et al. (2014)** | 0 (unknown) | 0 (not known) | 1 | 2 | 3 (Poor) |
| **19** | **Wilson et al. (2011)** | 2 | 2 | 2 | 1 | 7 (Good) |

1. Environmental measure as exposure variable which includes a set of built enviornemnt features, hence, controls for the impact of a number of attributes

2. Walkability measure as exposure variable which includes a set of built enviornemnt features, hence, controls for the impact of a number of attributes

**Rating**

(2=met, 1=partly met, 0=not met)

**Global rating:**

Good: 3 ratings “met” OR 2 ratings “met” and 2 ratings “partly met”

Fair: 2 ratings “met” OR 1 rating “met” and at least 2 ratings “parly met”

Poor: others (note: if the average score was between 2/1/0 (eg. 0.5), it was rounded up.)

**Criteria**

1. What is the response rate? 2=>60%, 1=41-60%, 0=<40% or not known
2. Is the sample surveyed representative of the population for whom the result will be generalised? 2=if stated that representative or comparison with population provided; 1=if some minor limitations; 0=not met or not known.
3. Are valid or reliable or standardised measures used for the outcome measure? 2=validated qustionnaire; 1=self-reported physical activity, 0=not met or not known
4. Were confounders controlled? 2=on individual and neighbourhood level, 1=on individual level; 0=not met or not known. Additional 0.5 points if control for self-selection.

**Maximum attainable 8.5**

Table 2 Quality assessment longitudinal and quasi experiments studies

| **Ref.** | **Publication** | **Are intervention/exposed and control/non exposed group similar?**  2=if stated  1=if some minor limitations  0=not met or not known or not applicable | **Are study participants adequately described (age, sex, baseline for outcomes)?**  2=if stated  1=if some minor limitations  0=not met or not known | **Outcome measures**  2=validated questionnare  1=self-reported  0=not met or not known | **Confounding**  2=on individual and neighbourhood built environment attributes  1=on individual level  0=not met or not known  0.5 additional if control for self-selction | **Follow up**  2=stated and compared with base line  1=stated and not compared with baseline  0=not known | **Global rating** |
| --- | --- | --- | --- | --- | --- | --- | --- |
| **7** | **Giles Corti et al. (2013) (quasi-experiment)** | 0 (not applicable) | 2 | 2 | 2.5 | 2 | 8.5 (Good) |
| **9** | **Knuiman et al. (2014) (quasi-experiment)** | 0 (not applicable) | 2 | 2 | 2.5 | 1 | 7.5 (Good) |
| **10** | **Shimura et al. (2012)** | 0 (not applicable) | 2 | 2 | 2^1^ | 1 | 7 (Good) |
| **11** | **Sugiyama et al. (2013)** | 0 (not applicable) | 2 (same participants for baseline and follow up) | 2 | 1 | 1 | 6 (Good) |

1. Walkability measure as exposure variable which includes a set of built enviornemnt features, hence, controls for the impact of a number of attributes

**Rating**

(2=met, 1=partly met, 0=not met)

**Global rating:**

Good: 3 ratings “met” OR 2 ratings “met” and 2 ratings “partly met”

Fair: 2 ratings “met” OR 1 rating “met” and at least 2 ratings “parly met”

Poor: others (note: if the average score was between 2/1/0 (eg. 0.5), it was rounded up.)

**Criteria**

1. Are intervention/exposed and control/non exposed group similar?
2. Are study participants adequately described (age, sex, baseline for outcomes)?
3. Are valid/reliable/standardized measures used for the outcome measure?
4. What is the follow-up?
5. Were confounders controlled?

**Maximum attainable:10**
